# Othello syndrome in Parkinson’s disease: a systematic review and report of a case series

**DOI:** 10.1007/s10072-021-05249-4

**Published:** 2021-05-12

**Authors:** Giovanna De Michele, Gianluigi Rosario Palmieri, Chiara Pane, Carmen Diletta Paola Dello Iacovo, Sandra Perillo, Francesco Saccà, Giuseppe De Michele, Anna De Rosa

**Affiliations:** Department of Neurosciences and Reproductive and Odontostomatological Sciences, Federico II University, Via Pansini 5, 80131 Naples, Italy

**Keywords:** Delusional jealousy, Dopamine agonists, Othello syndrome, Parkinson’s disease, Visual hallucinations, Psychosis

## Abstract

**Introduction:**

Psychosis in Parkinson’s disease (PD) is common and consists of hallucinations, illusions, and delusions. Among the latter, delusional jealousy, also named Othello syndrome (OS), might impair the quality of life of both patients and their partners. We aimed to perform a systematic review and report a series of PD patients presenting with OS.

**Methods:**

A systematic review research was performed in PubMed database, excluding non-English articles, single case reports, reviews and neuropathology articles, comments, and articles concerning OS associated with deep brain stimulation (DBS) and levodopa-carbidopa intestinal gel infusion. We also described eleven PD patients (9 M and 2 F) with OS, identified in a cohort of consecutive 153 patients, comparing them with eleven matched no OS (nOS) PD subjects taken from the same cohort.

**Results:**

We included eight articles (four case series and four cross-sectional studies). OS resulted more common among males than females. We did not find higher levodopa dose and levodopa equivalent dose for dopamine agonists and for all anti-parkinsonian drugs in our OS group. In our case series, OS patients showed visual hallucinations (*p*=0.001) and a trend to have depression (*p*=0.080) more frequently than nOS ones.

**Conclusions:**

OS is not a rare disorder in PD, probably due not only to abnormal dopaminergic stimulation but also to serotonergic dysfunction in biologically predisposed subjects. Visual hallucinations and other concomitant psychiatric diseases, in particular depression, might represent a risk factor for the OS development.

## Introduction

Parkinson’s disease (PD) represents the most common neurodegenerative disorders after Alzheimer’s disease [1]. The cardinal symptoms are bradykinesia, resting tremor, and muscle rigidity, but non-motor features are also frequent, including cognitive, psychiatric, gastrointestinal, urinary, cardiovascular, and sensory disorders [1]. The non-motor symptoms seem to be closely correlated to the spread and the progression of Lewy body pathology beyond the dopaminergic nigrostriatal pathway, involving the cortical and limbic regions, the non-motor midbrain nuclei, and the peripheral autonomic nervous system.

Psychosis in PD (PDP) has a lifetime prevalence of 47–60% and is characterized by minor phenomena, as “presence and passage hallucinations” (patients feel that someone or something indefinite is nearby or fleeting shadows pass by them), formed visual and other sensory modality hallucinations with or without insight, and delusions, which are fixed false beliefs [2, 3]. Risk factors for PDP development have consistently shown to be cognitive impairment, particularly attention, executive, and visuospatial skill dysfunction, REM behavior disorder (RBD), dopaminergic and anti-cholinergic treatment, and *Glucocerebrosidase* gene mutations, whereas association with older age and late onset of disease seems to be unconfirmed [3]. Delusion prevalence within PDP spectrum is estimated from 3 to 10% and results higher in PD dementia [3].  Delusion usually consists of feelings of guilt or sin, religious, grandiosity, reference, persecution, and jealousy themes [2]. Younger age, earlier onset of disease, higher frequency of impulse control disorders (ICD) and dopamine dysregulation syndrome, and lower rates of cognitive impairment have been reported in patients presenting with isolated delusions in comparison with those showing both delusions and hallucinations [4]. Delusional jealousy (DJ), also defined as Othello syndrome (OS), represents the most common type of delusion after persecution mania among PD patients [4], and seems to be mainly related to dopamine agonists (DA) and younger age, and often not associated with dementia, as usually observed for visual hallucinations (VH) [2, 3]. OS consists of a content-specific delusion whose theme is focused on betrayal and characterized by a range of irrational thoughts and emotions, together with concomitant unacceptable or extreme behavior [5]. OS patients base their suspicions of infidelity on unfounded evidence such as random events, coincidences, bits of conversation, and misplaced household items [6]. The disorder persists in the absence of any objective basis for suspicion, ordinary situations are misunderstood, and the partner’s actions are misinterpreted to the point of leading to an absolute conviction of repeated betrayal, associated with severe confusion and anxiety.

OS represents a dangerous and disabling condition, often causing agitation and aggressivity, and affecting the quality of life of patients and their families.

OS prevalence has been estimated to be 1.1% in psychiatric in-patients, particularly frequent in organic psychosis [7], whereas it is not well defined in PD. In addition to PD, OS has been also reported in other neurological disorders as stroke, brain trauma, brain tumors, encephalitis, multiple sclerosis, and normal pressure hydrocephalus [6]. Furthermore, DJ is associated with chronic alcoholism and recreational drug use, as cocaine and amphetamines [5]. OS related to PD and organic conditions is less organized and non-bizarre in comparison to that observed in schizophrenia [5].

So far, studies assessing OS in PD are few and limited by heterogeneity of the samples, differences in outcomes and evaluation tools, and availability of control groups. Our aim is to perform a systematic review to analyze the published literature and to describe motor and non-motor features in a personal series of PD patients with OS, in comparison with patients without OS (nOS) matched for gender, age at onset, and disease duration.

## Methods

A literature search was performed in June 2020 using the US National Library of Medicine National Institute of Health (https://pubmed.ncbi.nlm.nih.gov), selecting the articles having the following search terms in the title and/or abstract: “Parkinson’s Disease,” and “levodopa”, or “rotigotine”, “pramipexole”, “ropinirole”, “apomorphine”, “pergolide”, “cabergoline”, “dopamine agonists”, and “delusional jealousy” and/or “Othello syndrome”.

## Results

**Fig. 1:**
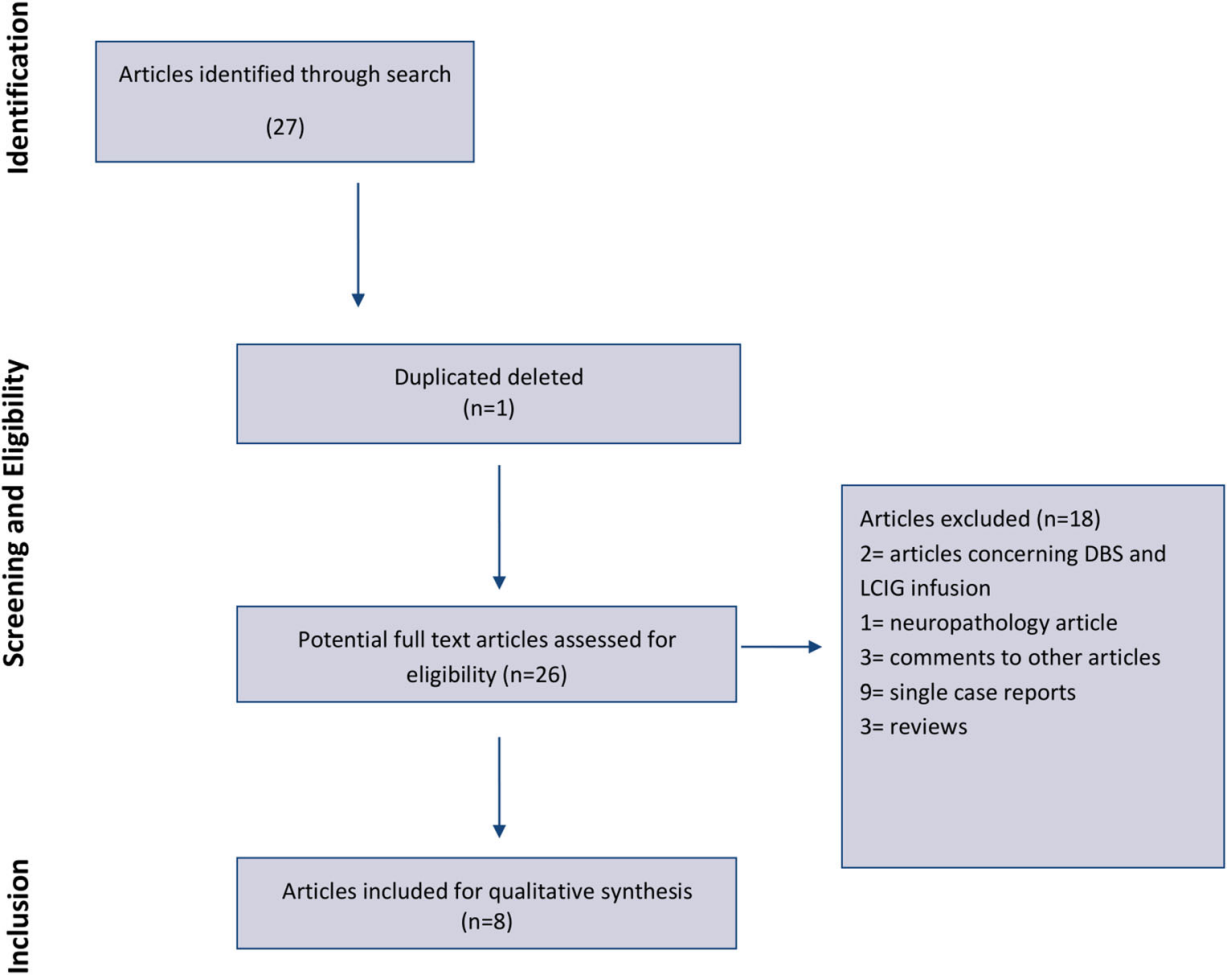
Diagram of the selection procedure to identify articles included in the systematic review

We retrieved 27 articles and included eight of them in our review (Fig. 1; Table 1). The reasons for exclusion were the following: articles concerning DBS and levodopa-carbidopa intestinal gel infusion (2), neuropathology article (1), comments to other articles (3), single case reports (9; three of them were in non-English language), reviews (3; one of them was in non-English language), and article partially duplicating previously published data (1). The selected articles included case series (4) and cross-sectional studies (4) (Fig. 1). Two studies described patients belonging to the same cohort [8, 9], but the last one reported psychiatric and neuropsychological features.

**Table 1:** Summary of reviewed studies

REF.	[8, 9]	[10]	[11]	[12]	[13]	[14]	[15]
No/M 60/45	20/14 M	5 /3 M	6/6 M	14/13 M	3/1 M	5/3 M	7/5M
PREV	2.5%	1.2%	1.06%	4%	NR	NR	NR
AO/A	53.5±9.0/63.7±7.6	48.8±8.6/NR	47.5 ±3.7/52.3±2.7	53.5±8.8/NR	52.9±9.5/67.6±10.3	46.8±8.8/NR	46.6 ±5.0/NR
OS onset	NR	NR	52.2±2.9	NR	65±11.1	56.4±8.76	53.7±7.0
UPDRS-HY	2.1*–2.1± 0.8	NR	16.8±1.8–2.7±0.3	NR	17.6±5.0–3	NR	NR
MCI/Dem	5 (25%)	NR	NR	NR	1 (33%)	0	4 (58%)
VH/OH	2 (10%)/NR	2 (40%)/NR	0/1 (16%)	NR/NR	3 (100%)/0	0/2 (40%)	NR/NR
Other delusions	NR	2 (40%)	NR	9 (64%)	2 (66%)	5 (100%)	NR
ICD	3 (15%)	5 (100%)	NR	NR	0	NR	7 (100%)
OPD	8 (40%)	NR	1 Paraphilic behaviors and Frotteurism	NR	0	NR	4 (58%)
DA	11 Pra, 8 Rop, 2 Cab	4 Rop, 1 Rot	2 Pra, 1 Rop, 2 Per	NR	1 Rop, 1 Cab+Rop, 1 Per	1 Apo, 1 Apo+Pra, 1 Pra, 2 Rop	5 Pra, 2 Rop
Measures	PPQ, Neuropsychiatry Inventory, UPDRS-III, HY	NR	UPDRS-III, HY	NR	UPDRS-III, HY	NR	CDR
Outcome^§^	5, need for addition of the neuroleptic in all	3	6, need for addition of the neuroleptic in all	NR	2, need for addition of the neuroleptic in 2	3, need for addition of the neuroleptic in 2	7

Abbreviations: *REF* reference; *No* number; *M* males; *PREV* prevalence; *NR* not reported; *AO* age at onset; *A* age at examination; *UPDRS* Unified Parkinson’s Disease Rating Scale section III; *HY* Hoehn & Yahr scale; *MCI* mild cognitive impairment; *Dem* dementia; *VH* Visual Hallucination; *OH* other hallucinations; *ICD* Impulsive Control Disorders; *OPD* other psychiatric disorders; *DA* dopaminergic agonists; *Pra* pramipexole; *Rop* ropinirole; *Per* pergolide; *LD* levodopa; *Apo* apomorphine; *Cab* cabergoline; *Rot* rotigotine; *PPQ* Parkinson Psychosis Questionnaire; *NPI* Neuropsychiatry Inventory; *CRD* Clinical Dementia Rating Scale

*not calculable

^§^Resolution/reduction after DA withdrawal/reduction

### Epidemiological and demographic data

OS was reported in 60 patients (45 M and 15 F) [8–15] (Table 1). The disorder was more common among males than females (3:1), frequently occurring in middle age. The overall prevalence was reported or obtainable in four studies only and resulted ranging between 1.06 and 4% [8, 10–12]. The mean age at PD onset was significantly lower among OS patients in comparison to nOS patients [12], ranging between 39 and 57 years [11, 13, 15], whereas the OS onset was between 49 and 77 years [11, 13–15] (Table 1). Familial history of psychiatric disorders was assessed in three studies [9, 11, 14] and was reported in nine out of 26 patients [9, 11].

### Clinical features

The clinical features have been summarized in Table 1. The clinical examination was performed by section III of the Unified Parkinson’s Disease Rating Scale (UPDRS) in 29 patients from three studies [9, 11, 13], resulting in individual scores from 13 to 23. The stage of disease was established according to Hoehn and Yahr (HY) scale in 29 patients from three studies [8, 11, 13] and the maximum individual value resulted 4 in one subject only, suggesting that most of OS patients presented with a moderate motor impairment. In particular, Poletti et al. reported a milder stage of disease, according to HY scale, among OS subjects in comparison with nOS patients with a comparable disease duration (*p*=0.020) [8].

One case of dementia was found among three OS patients in one study, whereas history of other previous psychiatric or personality disorders was not detected in any of them [13]. In another study, dementia, assessed by the Clinical Dementia Rating Scale, resulted very mild in two patients and mild in other two, whereas four cases presented with previous psychiatric disorders as depression, anxiety, and narcissistic personality disorder [15].

Poletti et al. reported dementia in five among 20 OS patients [8]. In a follow-up study, the same authors found that eight OS patients without dementia and three with cognitive impairment also suffered of other psychiatric disorders, including mood depression, anxiety, and bipolar disorder [9].

Foley et al. performed a full neuropsychological assessment, evaluating general intellectual functioning, non-verbal and verbal memory, language, visual perception, processing speed, and executive functions in five OS patients in comparison to five matched nOS ones [10]. They found significant abnormality of two executive tests measuring the response suppression ability and thinking time in the OS group.

ICD were observed in fifteen out of 32 OS patients [8–10, 15]. Hypersexuality was present in ten patients, pathological gambling in four, pathological shopping in five, and dopamine dysregulation syndrome in one.

Most of the included studies assessed the presence of other psychotic symptoms beyond OS, as multimodal hallucinations and other delusions [8–14]. Auditory hallucinations were reported by one patient, osmic hallucinations by two, VH by seven, and in all cases they had appeared before the OS onset [8, 10, 12–14]. Georgiev et al. also found persecutory delusions in concomitance of OS in four cases [14].

Neuroimaging findings were described in one study only: Brain MRI/CT was normal in four patients and showed right basal ganglia infarct in one [15].

### Treatment and outcome

Only one study reported levodopa dose (424.9±352.3 mg), DA (110.9±119.3 mg), and total levodopa dose equivalence (LEDD) (535 ± 366.0 mg) [12]. The authors found that DA treatment was more frequent among OS than among nOS patients.

DA was administered in 44 cases and levodopa in 39 (Table 1) [8–15]. Two patients took two different DA simultaneously [13, 14]. OS improvement or complete resolution was observed in 26 patients after DA withdrawal or dose decrease, and in one after selegiline withdrawal. One atypical neuroleptic (quetiapine or clozapine) was added in 30 cases to control the disorder and/or hallucinations [9–11, 13, 14].

## Limitations

Although OS in PD patients is not rarely detectable in clinical practice, so far articles on the topic are a few and mainly consist of single case reports. Furthermore, the studies conducted up to now are extremely heterogeneous, and include small samples of cases, and the results are contradictory. A direct comparison between the studies found in the literature seems to be complicated by the small sample size, the lack of control group and matching, and the absence/disparity of outcome measures.

## Case series

We identified eleven OS subjects (9 M and 2 F) in a series of 153 consecutive patients (51 F and 102 M), diagnosed as having PD according to the Movement Disorders Society criteria, participating in a screening study for *Glucocerebrosidase* gene variants, approved by the ethics committee. OS patients were compared with eleven nOS patients selected from the same cohort and matched by gender, age, and disease duration, obtaining information about the treatment and the presence of motor and non-motor fluctuations, dyskinesias, sleep, cognitive, neuropsychiatric, and autonomic disorders through semi-structured interviews performed during the screening and reported in the medical records. All patients were assessed by UPDRS-III, whereas the disease stage was evaluated by the HY scale. All subjects had also been screened for *LRRK2* gene mutations (G2019S and R1441C/G/H).

### Statistical analysis

Differences in non-parametric data between OS and nOS patients were analyzed using the Mann-Whitney *U* test. Qualitative data were compared by the Fisher’s exact test. A *p* value < 0.05 was considered statistically significant. The Statistical Package for the Social Sciences software for Windows (version 21.00, SPSS, Chicago, IL, USA) was used for the statistical analyses.

### Results

Demographic and clinical characteristics of both groups are shown in Table 2.

**Table 2:** Demographic and clinical features of OS and nOS patients

	Gender	Onset	DD	UPDRS	HY	Subtype	Side*	DA	LD	DA LEDD	Total^§^ LEDD
OS patients											
	M	60	12	18	2	AR	L	Rop	600	160	760
	M	63	3	20	1	TR	L	Pra	-	112	112
	M	64	6	28	3	TR	R	-	300	-	300
	M	53	17	25	2.5	TR	R	Pra	400	150	550
	M	47	10	22	2	TR	L	Rop	800	160	1060
	F	65	5	10	2	TR	L	Rop	-	120	120
	M	64	14	37	2.5	TR	R	Pra	300	150	450
	F	46	16	26	2	TR	R	Pra	750	200	950
	M	68	5	18	2	TR	L	Rot	600	120	820
	M	70	5	35	2.5	AR	L	-	400	-	400
	M	53	21	53	4	TR	R	Pra	800	300	1200
nOS patients											
	M	62	7	10	1	TR	L	Pra	200	0.75	375
	M	72	5	14	2	TR	L	Rop	420	50	470
	M	61	11	30	2	TR	R	Pra	600	150	750
	M	50	12	22	2	AR	L	Pra	450	300	750
	M	49	9	30	2.5	TR	L	Pra	400	75	475
	F	62	9	25	2.5	TR	L	-	500	-	500
	F	45	16	8	1.5	TR	R	Pra	300	300	600
	M	60	12	26	2.5	AR	L	Rot	700	120	820
	M	66	7	25	2	TR	L	Rop	100	120	320
	M	63	2	24	1	TR	R	Pra	-	300	300
	M	51	21	44	4	AR	R	Pra	1400	300	1700

Abbreviations: *OS* Othello syndrome; *DD* disease duration; *UPDRS* Unified Parkinson’s Disease Rating Scale section III: *HY* Hoehn and Yahr scale; *DA* dopaminergic agonists; *LD* levodopa (mg); *LEDD* levodopa dose equivalence (mg); *AR* akinetic-rigid form; *TR* tremulous form; *L* left; *R* right; *Rop* ropinirole; *Pra* pramipexole; *Rot* rotigotine

*More affected side

^§^Total LEDD also includes other anti-PD drugs (MAO-B and COMT inhibitors, amantadine, and anticholinergics)

We confirm that OS is more common among males (82%), but the overall prevalence in our series is 7.2%, higher than that previously reported. The mean age ± SD at OS onset is 67.7±4.8 years (range 60-73) and the disorder occurred both early and later during the course of disease. Most of the patients were in a mild or moderate stage of disease.

Other typology of delusions occurred in two OS patients, as persecutory and Capgras delusion, whereas eight of them also presented VH, with an overall prevalence higher than in reviewed articles. All patients showed OS simultaneously or within 4 years from the VH onset.

Six OS patients had mild cognitive impairment (MCI) and one dementia diagnosed according to established criteria, versus three MCI in the nOS group. ICD were complained by four patients; pathological gambling was reported by two subjects, compulsive eating by three, hypersexuality by two, and punding by one, whereas one nOS patient showed compulsive shopping.

Nine OS and ten nOS patients were treated with DA. All patients taking DA were on stable treatment and had not undergone a dose increment immediately before OS onset. DA and levodopa were administered in seven OS and nine nOS, only levodopa in two OS and one nOS case, and only DA in two OS and one nOS patient. The disorder significantly improved or disappeared after DA dose reduction or slow withdrawal in seven cases (1, 2, 4, 8, 9, 10, 11), and the administration of quetiapine was necessary in five cases (3, 5, 6, 7, 9), in addition to levodopa decrease in five of them (3, 5, 7, 9, 10).

We did not find any significant differences between the two groups for age at exam, disease duration, subtype (tremor-dominant or akinetic-rigid), more affected side, severity and stage of disease, levodopa dose, DA LEDD, total LEDD, typology of DA, presence of apathy, self-reported olfaction, apathy, sleep and autonomic disorders, MCI/dementia, ICD, motor and non-motor fluctuations, and dyskinesias (Table 3). The VH frequency was higher among OS than among nOS patients (*p*=0.001), and we found a trend for depression (*p*=0.080).

**Table 3:** Comparison between OS and nOS matched patients

	OS patients (*n* = 11)	nOS patients (*n* = 11)	*p* value
Gender	9M 2F	9M 2F	1.000
Last examination age (yrs)*	69.7±6.0	69.2±5.6	0.767
Disease duration (yrs)*	10.3±6.0	10.1±5.2	0.974
Subtype	9 TR (82%)-2 AK (18%)	8 TR (73%)-3 AK (27%)	1.000
Left side more affected	6 (54%)	7 (64%)	1.000
Hoehn and Yahr stage** 2 (1-4)^§^	One HY 1, five HY 2, three HY 2.5, one HY 3, one HY 4	Two HY 1, one HY 1.5, four HY 2, 3 HY 2.5, one HY 4	0.429
UPDRS-III*	26.5±11.7	23.4± 10.2	0.693
Levodopa dose (mg)*	450±289	461±374	0.817
DA LEDD (mg) *	133.8±84	162.7±115.6	0.868
Total LEDD (mg)*	611.1±372.8	641.8±393.2	0.974
MCI/dementia	6 (54%)/1 (9%)	3 (27%)/0	0.387/1.000
ICD	4 (36%)	1 (9%)	0.310
Depression	7 (64%)	2 (18%)	0.080
Visual hallucinations	8 (73%)	0	**0.001**
Other delusions	2 (18%)	0	0.476
Anxiety	5 (45%)	2 (18%)	0.361
Dysautonomia	3 (27%)	4 (36%)	1.000
RBD	9 (82%)	7 (54%)	0.635
Psychosis	7 (64%)	8 (73%)	1.000
Motor fluctuations	7 (64)%	7 (64%)	1.000
Onset motor fluctuations (yrs)*	65.3±6.8	63.7±5.6	0.892
Non-motor fluctuations	0	2 (18%)	0.476
Dyskinesias	4 (36%)	2 (18%)	0.635
Onset dyskinesias (yrs)*	62±7.2	62±1.4	0.687
Apathy	3 (27%)	0	0.214

Significant values are in bold. Differences in non-parametric data between OS and nOS patients were analyzed using the Mann-Whitney *U* test. Qualitative data were compared by the Fisher’s exact test

Abbreviations: *OS* Othello syndrome; *yrs* years; *TR* tremulous form; *AR* akinetic-rigid form; *UPDRS* Unified Parkinson’s Disease Rating Scale section III; *DA* dopaminergic agonists; *LEDD* levodopa dose equivalence; *MCI* mild cognitive impairment; *ICD* impulsive control disorders; *RBD* REM behavior disorders

*Values are means (SD)

**Median

^§^Range

## Discussion

Psychosis is a non-motor, disabling and sometimes neglected feature in PD, characterized by multimodal hallucinations, illusions, and delusions. Delusional disorder prevalence is estimated to be about 5% [16].

Here, we aimed to review critically the published literature, attempting to clarify several aspects of OS in PD. Furthermore, we presented a series of OS cases comparing them with nOS PD subjects in relation with both motor and non-motor features, with the aim to highlight any clinical markers that might characterize these patients.

The systematic review and our study results show that OS is more common among males than in females. In our sample, the prevalence was 7.2%, resulting higher than that previously reported in PD patients, and the disturbance occurred simultaneously or within 4 years from the VH onset. Our study and the reviewed papers show that most OS patients present the disorder in the initial-middle stage of disease, characterized by mild/moderate motor impairment. As previously observed, we did not find a higher prevalence of MCI/dementia and ICD among OS, whereas VH significantly resulted more frequent in the OS sample.

The pathophysiological mechanisms underlying the OS development in PD patients are unclear and poorly understood, and although a number of cerebral disorders might lead to this disorder, a specific lesion or degeneration area pattern has not been defined. VH and OS might partially share a common neurodegenerative process involving frontal and temporal cortex. In particular, the frontal lobe preeminently seems to promote the ability to integrate and correct perceptual distortions by using new information to adopt appropriate behaviors. Frontal dysfunction may cause the impossibility to resolve and handle contradictory and conflicting evidence, impairing self-correcting and leading to false beliefs [6, 17]. MRI study in OS patients affected with neurodegenerative disorders, including PD, showed higher gray matter loss predominantly in the dorsolateral frontal lobes, particularly in the superior frontal gyri, and in the right posterior lateral temporal lobe, in comparison to matched neurodegenerative patients without delusions [17]. Furthermore, significant cortical atrophy has been found in the bilateral dorsolateral prefrontal cortex and left fusiform gyrus, beyond other limbic, parietal, and occipital regions, in PD patients with VH compared to those without VH [18].

A relationship between psychosis in PD and other psychiatric disorders, in particular depression and anxiety, has been also reported, even in the early stage of disease [6, 8, 9, 16, 19]. Interestingly, we find that OS subjects showed a trend to have depression more frequently that the nOS group. This finding may be explained by the progressive serotoninergic degeneration described in PD, not only leading to depression and anxiety, but also resulting in compensatory 5HT2A receptor upregulation, which is observed preferentially in the prefrontal and visual cortices of PD patients with psychosis [20]. So, OS could also be associated with stimulation of 5HT2 receptors, in addition to dopaminergic ones, as already established for the pathogenesis of hallucinations [20]. Indeed, atypical antipsychotics with relevant 5HT2A blockade activity, as clozapine, and potent selective 5-HT2A inverse agonists, as pimavanserin, showed clinical trial evidence of efficacy in PDP management [3].

This hypothesis may partially explain why in our study DA typology, levodopa dose, DA and total LEDD were not significantly different between the OS and nOS groups, suggesting that other mechanisms underlie the disorder. Moreover, the adjustment of DA therapy did not improve or resulted in partial resolution of OS in most of cases [9, 10, 13, 14]. Although DA are traditionally considered to play a causal role for PD delusions [8] and psychosis in general, a clear association has not been confirmed, as these disorders are also observed in drug-naïve or treated with levodopa only patients, and the risk to develop them is not related to LEDD [21]. The high frequency of psychiatric diseases among OS patients [11, 15] suggests that the DA may act as a “modifier” or a risk factor in vulnerable subjects with premorbid personality. So, it is likely that “overstimulation” or sensitization of the mesolimbic dopaminergic receptors, rather than the type or dose of DA, concurrently with 5HT2 receptor upregulation, might favor OS development in subjects presenting with biological predisposition to psychiatric disorders [22], as early onset PD (EOPD) patients, consequently leading to abnormal perception and mental representation of environmental inputs, and inappropriate behavior.

OS may be observed at any stage, despite a DA treatment at low dose and for a short period; however, it is not usually associated with cognitive impairment when it occurs in early stage of disease and in EOPD [9]. Indeed, DJ associated with dementia and hallucinations more frequently occurs in advanced stage of disease [9]. This finding may be partially explained by the neuronal loss in the cholinergic nucleus basalis of Meynert observed in late PD with cognitive decline. Furthermore, cholinesterase inhibitor efficacy on hallucinations, delusions, and agitation is widely proven in both PD with dementia and Lewy body disease [23].

Actually, a classification of PDP subtypes according to the progression of neuronal degeneration and sequential neurotransmitters involvement has been proposed [24]. Early in disease, patients mostly experience minor psychotic symptoms such as illusions or presence/passage hallucinations, due to dopaminergic stimulation [2]. As PD progresses, due to the addition of serotonergic and cholinergic dysfunction, affective disorders, delusions, formed visual, and other modality hallucinations occur, often associated with cognitive decline [24].

The role played by the noradrenergic (NE) system in the pathogenesis of the psychosis in schizophrenia and agitation/aggression in Alzheimer’s disease is known [25]. NE system dysfunction observed in PD has been shown to contribute to non-motor symptoms, as depression/anxiety, cognitive decline, sleep impairment, and cardiovascular disorders [26], but it has not been thoroughly investigated in psychosis pathogenesis. Locus coeruleus, considered the main NE nucleus in the central nervous system, modulates the release of dopamine in the projections to the striatum, influencing the activity of substantia nigra pars compacta, ventral tegmental area, and prefrontal cortex [25, 27]. Furthermore, NE and dopamine release in the hypothalamus and forebrain regulates the responses to stress [25], so the imbalance of both neurotransmitters, interacting with each other, might affect the potential behavioral reactions to multimodal inputs and lead to delusions.

In conclusion, OS is not rare in PD patients, especially among men, and can lead to severe impairment of quality of life, negative emotional reactions, and aggressive and dangerous behaviors. The occurrence of VH and depression might support the physicians to detect early and adequately manage OS, which is often not spontaneously reported by the patients and their partners.
